# Estimation of HTLV-1 vertical transmission cases in Brazil per annum

**DOI:** 10.1371/journal.pntd.0006913

**Published:** 2018-11-12

**Authors:** Carolina Rosadas, Bassit Malik, Graham P. Taylor, Marzia Puccioni-Sohler

**Affiliations:** 1 Imperial College London, London, United Kingdom; 2 Universidade Estácio, Rio de Janeiro, Brazil; 3 Universidade Federal do Rio de Janeiro, Rio de Janeiro, Brazil; 4 Universidade Federal do Estado Rio de Janeiro, Rio de Janeiro, Brazil; Institute for Disease Modeling, UNITED STATES

## Abstract

**Background:**

Brazil has at least 800,000 HTLV-1 infected individuals. HTLV-1 can be transmitted via sexual intercourse, contact with blood and from mother to child, mainly by breastfeeding. Treatments for the high morbidity/mortality associated diseases (ATL and HAM/TSP) are limited, therefore, infection prevention is of utmost importance. However, antenatal screening is not routinely performed in Brazil. A lack of data regarding the number of individuals infected via breastfeeding impairs the development of government policies. The objective is to estimate the number of HTLV-1 infections occurring annually due to mother to child transmission (MTCT) in Brazil, nationally and regionally.

**Methodology:**

To estimate HTLV-1 MTCT in Brazil the following variables are modelled: number of births, prevalence of HTLV-1 infection in pregnant women, breastfeeding duration rate and transmission risk according to breastfeeding period. The number of cases of HAM/TSP and ATL attributable to MTCT are also estimated.

**Principal findings:**

In 2008, there were 2,934,828 live births in Brazil. HTLV prevalence in pregnant women in Brazil ranges between 0.1–1.05% by region. An estimated 16,548 HTLV-1 infected women are pregnant each year. According to the breastfeeding pattern and HTLV-1 prevalence of each region there are an estimated 3,024 new cases of HTLV-1 infection due to MTCT annually of which 2,610 are preventable through infant feeding advice. These 3,024 transmissions will result in 120–604 cases of ATL and 8–272 of HAM/TSP. North-East region comprises the high number of MTCT cases, followed by South-East.

**Conclusions/significance:**

A high number of new HTLV-1 infections due to MTCT occur every year in Brazil. Antenatal screening and avoiding breastfeeding are essential to prevent subsequent development of HTLV-1-associated diseases.

## Author summary

The human T-cell lymphotropic virus type 1 (HTLV-1) causes lifelong infection in humans. Although most infected individuals remain asymptomatic HTLV-1 causes severe disease in at least 5% of carriers. In the present study we estimate the number of HTLV-1 infections that occur annually due to mother to child transmission (MTCT) in Brazil and within its regions. The high number of estimated cases due to MTCT (3,024) which will give rise in adult life to HTLV-1associated diseases suggest the importance of performing HTLV-1 antenatal screening in Brazil to reduce the dissemination of this infection and to thus prevent a blood cancer to which most individuals succumb within 8 months of presentation and in others a disabling disease of the spinal cord.

## Introduction

The human T-cell lymphotropic virus type 1 (HTLV-1) infects at least 5–10 million individuals throughout the world. Brazil has approximately 800,000 carriers [1]. This virus can be transmitted via sexual intercourse, contact with infected blood and from mother to child, mainly through breastfeeding[1]. Most infected individuals (>90%) remain lifelong asymptomatic carriers but are infectious, perpetuating HTLV-1 transmission. However, the diseases associated with this infection, such as HTLV-1-associated myelopathy / tropical spastic paraparesis (HAM/TSP) and adult T cell leukaemia (ATL) have high morbidity and mortality[1–3]. The life-expectancy of ATL at 8 months is one of the shortest of all malignancies. Half of all patients who develop HAM/TSP become wheelchair dependent. Treatment for these conditions remain limited.

HTLV-1 infection may be detected using screening tests such as enzyme-linked immunosorbent assay (ELISA). However, confirmatory and typing tests are mandatory to conclude the diagnosis. Western blot (WB) and the HTLV-1 proviral DNA detection by polymerase chain reaction (PCR) are considered confirmatory tests [4–6].

To reduce HTLV-1 transmission in Brazil, since 1993 blood donations are tested before transfusion[7]. However, antenatal screening is not routinely performed by the national health system of Brazil (Sistema Único de Saúde, SUS). Mother-to-child transmission (MTCT) is responsible for maintaining the virus for several generations in the same family [8,9].

Several studies have demonstrated that a reduction in the number of mothers breast-feeding and a shortening of the breast-feeding period decreased the MTCT by about 80% [10,11]. This is evident in Japan, which implemented a successful program to prevent HTLV-1 vertical transmission in some regions [12] and now has a national programme.

Lack of data regarding the number of individuals infected via breastfeeding impairs the development and implementation of government policies. In 2016, for example, in Brazil, there were 87 new diagnoses of HIV infection reported in children under 5 years[13]. However, this number is not known for HTLV infection.

Our aim is to estimate the number of HTLV-1 infections that occur annually due to mother to child transmission (MTCT) in Brazil.

## Methods

For the estimation of the number of HTLV-1 cases due to MTCT per year in Brazil, the following variables were used: number of births in Brazil and its regions, prevalence of HTLV-1 infection in pregnant women in the country, breastfeeding duration rate and transmission risk according to breastfeeding period. We also estimated the number of HAM/TSP and ATL cases due to MTCT according to the lifetime risk of each disease. The number of births in Brazil and the breastfeeding duration rate were acquired from Health Ministry of Brazil data.

### HTLV prevalence in pregnant woman from Brazil

To determine the prevalence of HTLV-1 infection in pregnant women in Brazil, a literature review was performed according to recommendations established by Preferred Reporting Items for Systematic Reviews and Meta-Analysis (PRISMA)[14]. The search terms “HTLV”, “prevalence” and “Brazil” were used in Pubmed, Scopus and Scielo databases. The search was conducted in October 2017 and there was no restriction regarding the publication year. Only articles written in English, Spanish or Portuguese were considered. Books or book chapters, comments, editorials and reviews were not included.

The following inclusion criteria were: 1) studied population includes Brazilian pregnant women; 2) it provides measures of prevalence; 3) studies used confirmatory tests for HTLV-1 infection, such as WB or molecular assays, such as PCR. The exclusion criteria were: 1) studies that did not provide prevalence; 2) studied population was not pregnant women; 3) pregnant women were not from Brazil; 4) the study performed only screening test for HTLV infection.

For this systematic review no protocol was registered, and no quality scoring system was applied.

Data regarding the number of analysed samples, year of study publication, HTLV-1 test method, Brazilian state, prevalence of HTLV in pregnant women were extracted from the selected studies.

### Breastfeeding duration rates in Brazil

In 2008, a large study regarding the prevalence of breastfeeding within Brazil was performed by Brazilian Health Ministry. In this study, the prevalence of breastfeeding was determined from 30 days until 1 year after birth and it included analysis per country region and by Brazilian state [15]. These data were acquired and used in the present study (Table 1).

**Table 1:** Probabilities of children being breastfed according to age in days in different Brazil’s regions according to the Brazilian Ministry of Health (Ministério da Saúde do Brasil, 2009). [15].

Brazil's Region	Days						
	30	60	90	120	180	270	365
North	95.8	94.7	93.4	91.9	87.7	78	63.1
North-East	90.6	88.6	86.2	83.5	76.7	63.4	46.8
Mid-West	93.8	92.3	90.4	88.2	82.3	69.4	51.7
South-East	90	87.6	84.7	81.3	72.9	56.7	37.9
South	89.4	86.9	84	80.6	72.1	55.9	37.9

### Estimation of HTLV-1 Mother-to-Child transmission cases

**Fig 1:**
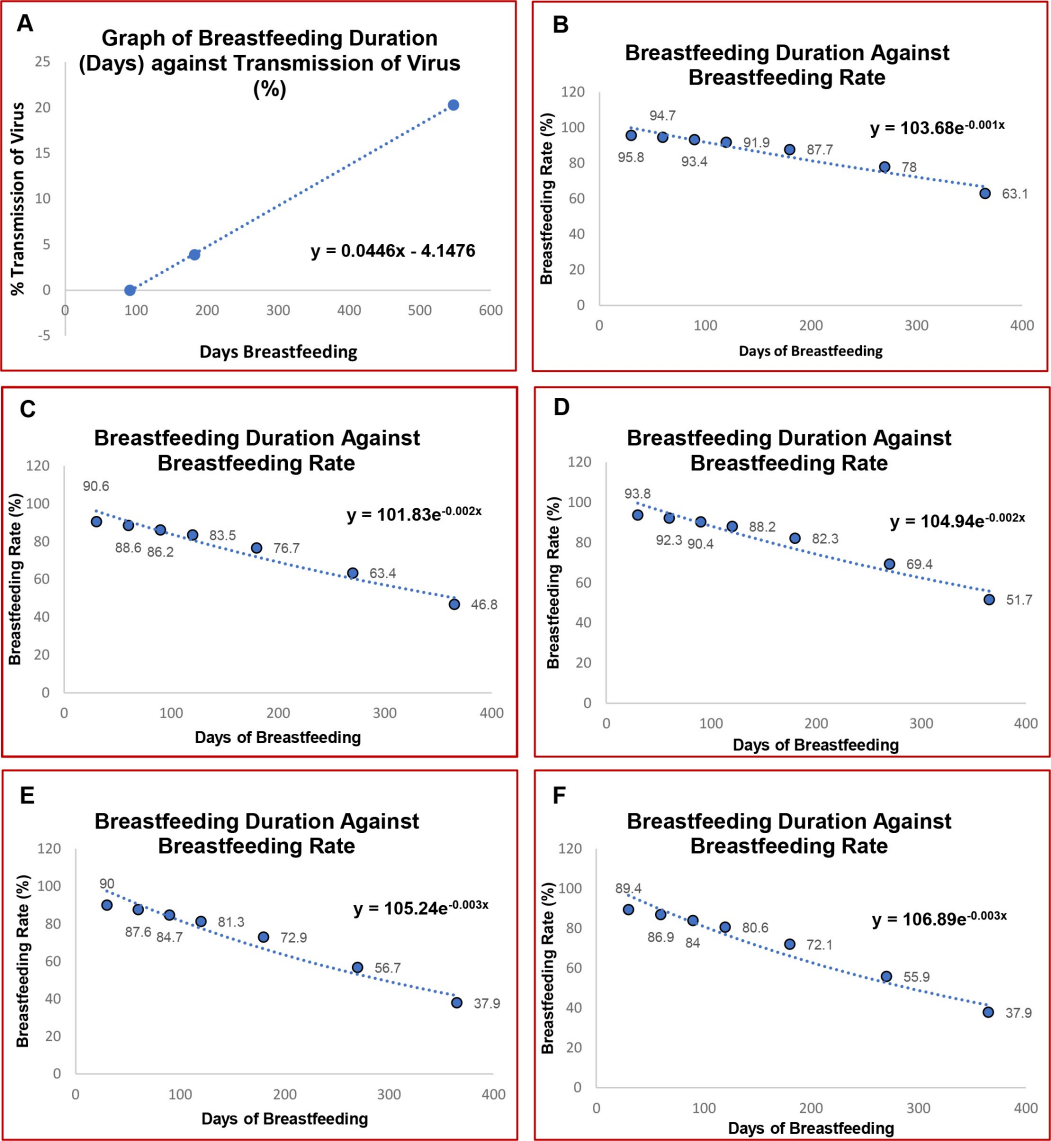
Estimation of HTLV-1 mother-to-child transmissions cases in Brazil. **A)** Model used to calculate the number of breastfeeding women over time in Brazil: Breastfeeding duration (days) against HTLV-1 transmission; **B-F)** Model used to evaluate the HTLV-1 transmission risk according to breastfeeding duration rates in the different Brazilian regions: **B)** North; **C)** North East; **D)** Midwest; **E)** South East; **F)** South.

To estimate HTLV-1 MTCT cases in Brazil we used the methodology described in Malik et al. 2018. The HTLV-1 transmission risk according to breastfeeding period assumed were those reported by Takezaki and colleagues [16]. A line of best fit was applied, and an equation derived from which come the estimates of transmission for 3 monthly increments (Fig 1). The transmission risks used in the manuscript were: 1.5 months–zero risk; 4.5 months—1.9%; 7.5m - 6.03%; 10.5m - 10.1%; 13.5m - 14.16%; 16.5m - 18.24%; 19.5m - 22.3%; 22.5m - 26.3%; 25.5m - 30% and up to 36m - 44.69% (Fig 1). These estimates are consistent with data from the region including studies from French Guyana [17] with a transmission rate of 10.6% in children breast-fed for a mean of 12 months and Jamaica [18] with a transmission of 32% who breast-fed for longer than 12 months. Finally, a very recent study from Brazil [19], demonstrated that the rate of transmission was 23.8% on long term breastfeeding (> 12 months) and 50% when breastfeeding duration was 24–36 months. These data plus the estimate from Takahashi that breast-feeding for less than 6 months was associated with 4.4% transmission (in Japan) leads us to believe that the attributed risk that we have used is consistent with all published data and errs towards a more conservative end of the range. As the literature indicates, up to three months breastfeeding confers zero risk of HTLV-1 transmission due to breast-feeding[13].

In a similar process, by retrieving published data on breastfeeding rates against time for each region of Brazil [12] (Table 1), a line of best fit was derived using Excel. The resulting exponential decay equation was used to extrapolate breastfeeding rates for a period of 0 to 3 years. Three years was deemed the maximum breastfeeding duration (Fig 1).

By multiplying the number of breastfeeding women in three-month periods from 0 to 3 years, midpoint viral transmission rate for the respective period and the seroprevalence of HTLV-1 amongst pregnant women in the population (Table 2); the number of HTLV-1 mother to child transmission cases from breastfeeding was determined.

**Table 2:** Summary of the selected studies for the systematic review regarding the HTLV-1 prevalence in pregnant women from Brazil.

Author	Year	State	Region	Prevalence	Screening method	n
Moura	2015	Alagoas	North-East	0.2	ELISA and WB	54,813
Monteiro	2014	Rio de Janeiro	South-East	0.66	CMIA and WB	1,204
Boa-sorte	2014	Bahia	North-East	0.14	Dried blood spot	692
Mello	2014	Bahia	North-East	1.05	ELISA, WB and PCR	2,766
Sequeira	2012	Pará	North	0.3	ELISA and WB	13,382
Guimarães de Souza	2012	Maranhão	North-East	0.3	ELISA (2), WB and PCR	2,044
Machado Filho	2010	Amazonas	North	0	ELISA and PCR	674
Magalhães	2008	Bahia	North-East	0.98	ELISA, WB, PCR	408
Dal Fabbro	2008	Mato Grosso do Sul	Mid-West	0.13	ELISA, WB and PCR	116,689
Figueiró Filho	2007	Mato Grosso do Sul	Mid-West	0.1	ELISA PCR (68%)	32,512
Oliveira	2006	Goiás	Mid-West	0,1	ELISA and PCR	15,485
Neto	2004	São Paulo	South-East	0.1	ELISA and WB	913
Bittencourt	2001	Bahia	North-East	0.84	ELISA, WB and PCR	6,754
Broutet	1996	Ceará	North-East	0.12	ELISA and WB	814
Santos	1995	Bahia	North-East	0.88	ELISA and WB	1,024

ELISA: Enzyme linked immunosorbent assay; WB: Western Blot; PCR: Polymerase chain reaction.

For completeness, a 2.5 percent risk of mother to child transmission from sources other than breastfeeding is incorporated in the analysis [13]. This was calculated by multiplying 2.5 percent with the product of the number of births annually multiplied by the seroprevalence of HTLV-1 amongst pregnant women (Fig 1).

### Estimation of ATL and HAM/TSP risk

HAM/TSP and ATL development rate comprises the proportion of individuals who develop those diseases following HTLV-1 infection. The lifetime risk of HAM varies between 0.25% to 3%. However, in Brazil, some studies showed a high risk for HAM/TSP onset [20]. The incidence in Tanajura et al. (2015) of 1.47% in 414 cases over a mean of 3 years equates to a 9% life-time risk. As it is not known whether infection in infancy or adult life has any impact on this risk, lifetime risks of 0.25%, 3% and 9% were imputed. For analysis purpose, ATL development rate was assumed to be 4%-20%, according to previous studies. While the ATL overall rate is estimated to be approximately 4% among all HTLV-1 infected individuals, it is known that ATL development is associated with childhood infection. Considering also that up to 85% of HTLV infections occurs in adult life, the risk of ATL development after vertical transmission should be adjusted, therefore, we considered a scenario with an upper limit of 20%. There are no data regarding ATL incidence in Brazil.

## Results

**Fig 2:**
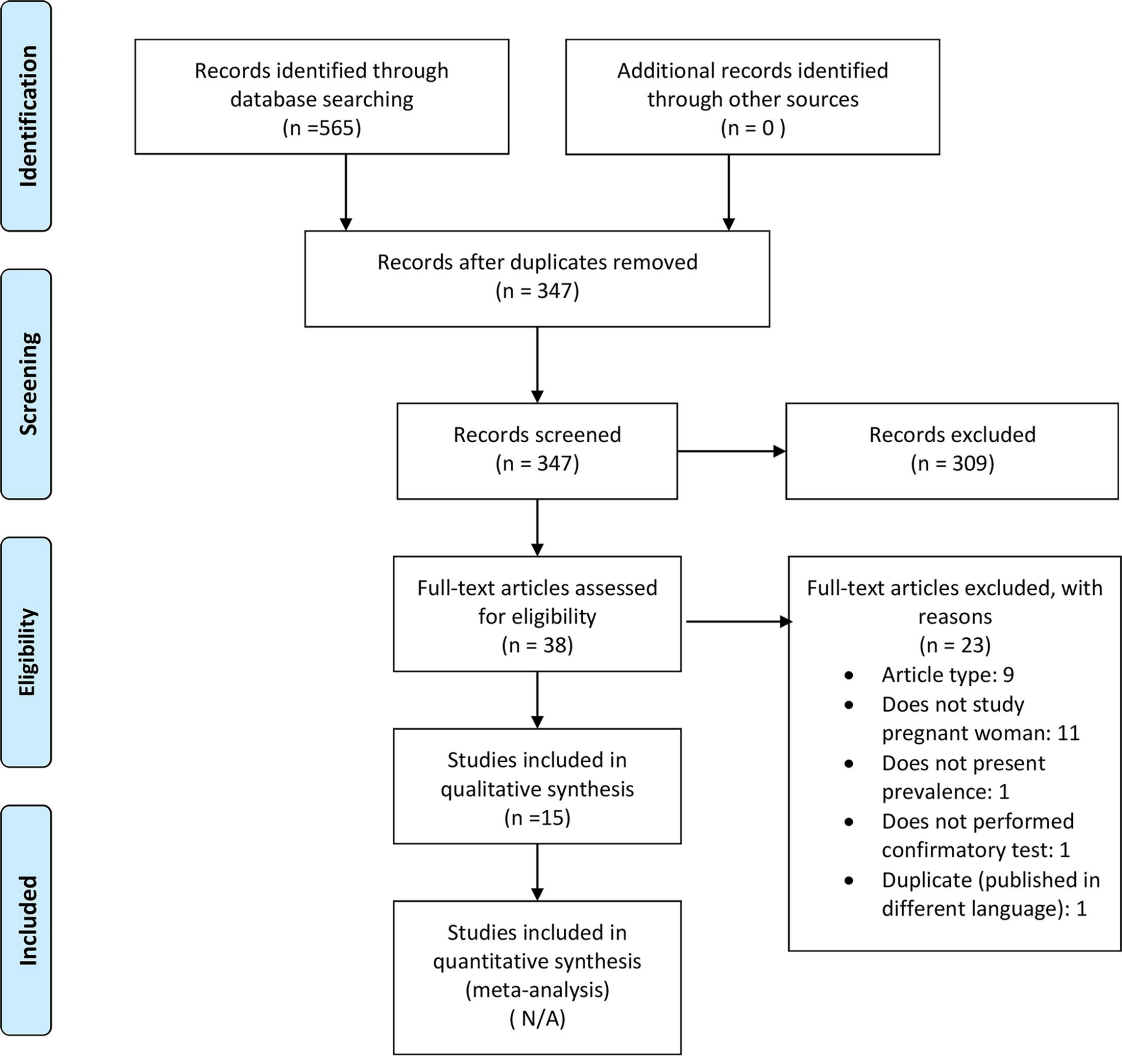
PRISMA flow chart for study selection. The flow diagram indicates the numbers of studies reviewed in preparation of the current systematic review of HTLV prevalence in pregnant woman from Brazil. Adapted from [14].

**Fig 3:**
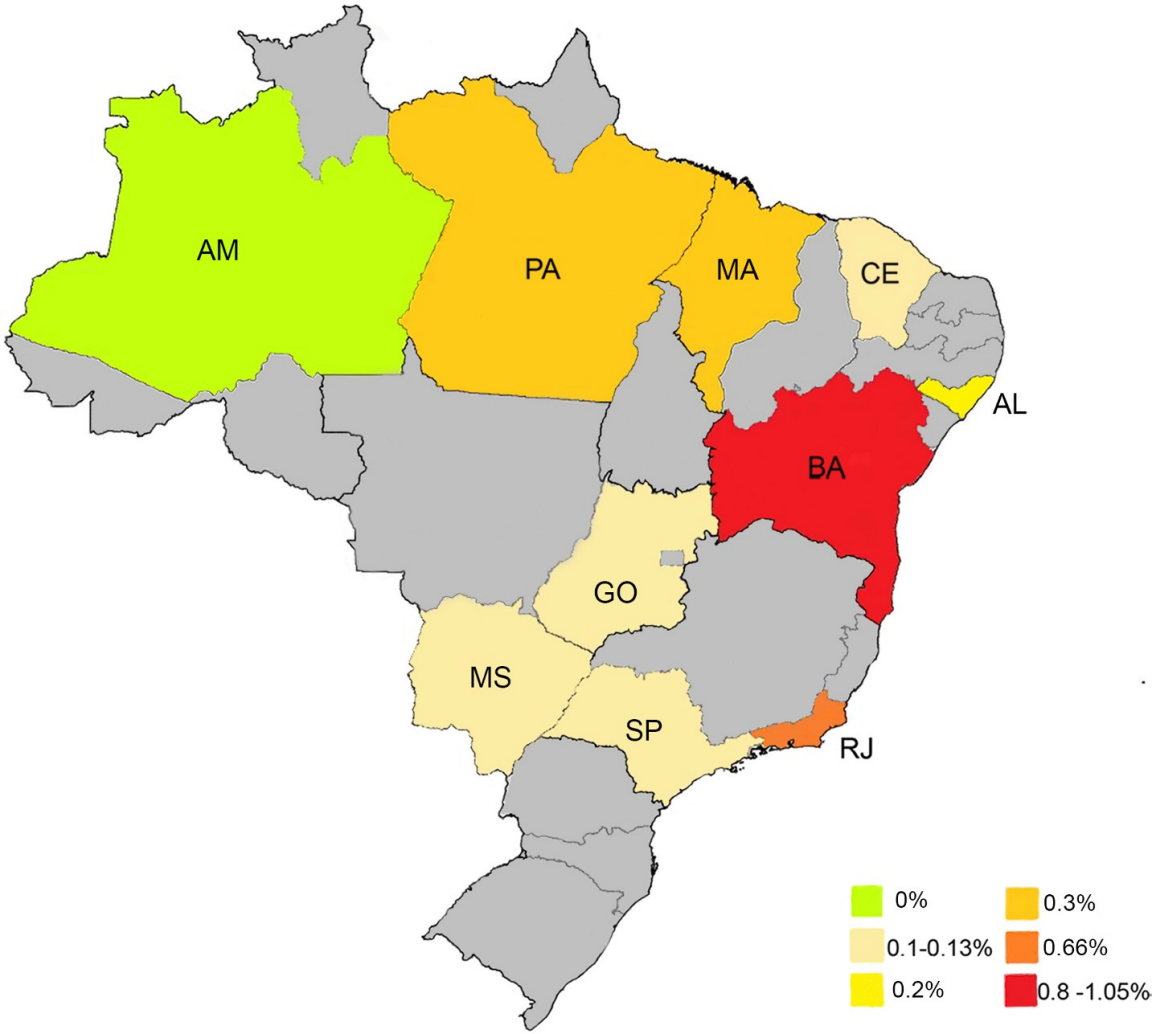
Representative map of HTLV-1 prevalence in pregnant women in different Brazilians states. The map was created with Paint 3D Windows and Adobe Photoshop CC 2018, using the open source map available at https://commons.wikimedia.org/wiki/Atlas_of_the_world.

The systematic review regarding HTLV prevalence in pregnant woman from Brazil identified 565 studies. Fig 2 represents the flow chart for study selection. Table 2 shows the included studies and the main findings [21–35]. The prevalence of HTLV-1 infection in pregnant women from Brazil varied between 0–1.05%. Due to the high diversity in HTLV prevalence observed between different regions of Brazil (Fig 3), we estimated the number of cases due to MTCT for each region. For this analysis, studies with less than 1000 individuals were excluded. For the south region, we assume the lowest HTLV prevalence observed among the different states in the country (0.1%), since there are no data available regarding the HTLV prevalence in pregnant women in this region. Considering the data from each of Brazil`s regions, we estimate that there are 16,548 pregnant women in Brazil infected with HTLV-1 each year (Table 3). However, if we just assume the highest and lowest HTLV-1 prevalence reported in pregnant women in Brazil (0.1–1.05%), the estimated number varies between 2,935–30,815 infected pregnant women/year.

**Table 3:** Estimated number of HTLV-1 infection, HAM/TSP and ATL cases due vertical transmission per year in Brazil and its regions.

Region	HTLV prevalence in pregnant women	Number of births (2008)	Number of infected pregnant women	HTLV infection due to MTCT per year			HAM/TSP 0.25–9%	ATL 4–20%
	%			Breastfeeding	Delivery	Total		
North	0.3	321,998	966	233	24	257	1–23	10–51
North-East	0.84	888,268	7,461	1,257	187	1,444	4–130	58–289
Mid-West	0.13	222,658	289	55	7	62	0–6	2–12
South-East	0.66	1,130,407	7,461	1,012	187	1,199	3–108	48–240
South	0.1*	371,497	371	53	9	62	0–6	2–12
Brazil		2,934,828	16,548	2,610	414	3,024	8–272	120–604

*assumed prevalence

In 2008, there were 2,934,828 births in Brazil. A summary of the findings for each region of Brazil is presented in Table 3. The North-East region contributes with the high number of HTLV-1 MTCT cases, followed by South-East region. Factoring in breast-feeding patterns a total of 2,610 new HTLV-1 infections per year through breastfeeding is estimated in the country. Moreover, 414 new vertical infections should occur annually through delivery and could not be avoided using breast milk substitutes. Therefore, the estimated total number of cases of HTLV-1 infections due to MTCT in Brazil is 3,024 per annum.

Regarding HTLV associated diseases, supposing 4–20% and 3% (0.25–9%) the risk of ATL and HAM/TSP development respectively, 120–604 ATL and 91 cases (8–272) HAM/TSP cases should occur in the future due to MTCT each year.

## Discussion

HTLV-1 infection and its associated diseases remain neglected [36]. While ATL is associated with high mortality and poor outcome, HAM/TSP is a disabling chronic disease which causes an important impairment in the patient`s quality of life. There is no cure available, only symptomatic treatment, increasing the costs to the health system and causing a negative impact on the patient`s life. Therefore, prevention is the best way to control the disease. For the implementation of effective measures to control the virus transmission, it is necessary to understand the scenario of each location. The determination of the number of new cases that occur per year due to mother to child transmission in Brazil is essential and can serve as a guide for the development and implementation of public health policies aimed at controlling this infection.

To estimate the number of new cases of HTLV infections due to MTCT in Brazil we used the transmission risk rates observed in Japan as they are very robust. A recent paper from Brazil, obtained very similar rates of those that were used, confirming that they are suitable for this study. Different factors, such as HTLV proviral load and coinfections can influence the MTCT risk. For example, in Peru, *Strongyloides* infection results in an increased rate of MTCT (31%) [37,38]. Early introduction of mixed feeding may also change the level of transmission. However, it is important to note that early introduction of breastfeeding is also a reality in São Paulo [15], where the study of Paiva et al (2018)[19] was conducted and observed transmission rates similar to those that were used in this study. Therefore, it is unlikely that will influence the predicted transmission rates.

The present analysis indicates that a large number of new cases of HTLV infections due to mother to child transmission in Brazil occur each year. When comparing with other diseases that are currently included in prenatal and neonatal screening, such as HIV, the size of the problem becomes more evident. Beside this, we also calculate that antenatal screening to identify infected mothers who would be advised regarding the risk of transmission and associated diseases and potentially to avoid or limit breastfeeding, would lead to the prevention of 104–522 cases of ATL and 6–235 cases of HAM/TSP which result from vertical transmission each year (excluding 16–82 ATL and 2–37 HAM/TSP cases, that are considered associated with the residual transmission). These diseases present high morbidity and mortality. However, even apparently asymptomatic infection has a negative impact on the life of HTLV-1 seropositive patients. Decreased quality of life, increased incidence of depression and anxiety among asymptomatic individuals diagnosed with HTLV infection has been demonstrated and are not necessarily a consequence of being informed of the diagnosis [39–41]. In Brazil, this can be exacerbated by the lack of care in public health that HTLV infected patients need to face as clearly reported by Zihlmann and colleagues (2012) [42].

In this study, to estimate the number of individuals that will develop clinical manifestations resulting from HTLV infection we used the range described in the literature. The risk of HAM/TSP development after vertical transmission is not known. However, there are many reports that HAM/TSP can occur after mother to child transmission [43–48]. Although the rate of HAM/TSP that seems more applicable for the country is 3%, in this study a range was used, includging the 0.25% calculated for Japan [49]. Recent studies indicate that Brazil may have a higher percentage of HAM/TSP and other neurological diseases among infected individuals [20]. In fact, Tanajura and colleagues (2015) followed a cohort of asymptomatic individuals from Brazil for up to eight years reported a high incidence of neurological disorders (up to 30%) and 1.5% incidence after 3 years. In the other hand, there are limited data on ATL in Brazil [44]. Therefore, we used the ATL life-time risk of all carriers (4%) as the conservative estimation. However, it is recognised that only those carriers who were infected during the perinatal and infancy period are at risk of ATL and is also known that approximately 80% of HTLV-1 infections are acquired in adult life. Therefore, the risk that infected infants will at some point in their adult life develop ATL should be as higher as 20% [50]. The present study also did not consider other clinical manifestation that can affect HTLV-1 infected individuals, such as infective dermatitis, uveitis, Sjögren's syndrome, Hashimoto's thyroiditis, and Graves' disease [6,51] or the impact of HTLV-1 infection on other infections, most notably Strongyloidiasis, tuberculosis and Hepatitis C.

Antenatal screening would also detect, and with the same intervention could also prevent mother to child transmission of, HTLV-2. This virus type was not included in the study, since the data regarding its prevalence are scarce, especially in pregnant women.

It is also worth noting that, preventing the occurrence of a single new case, it is possible to inhibit the transmission chain of this individual, blocking virus maintenance for generations and intra-family dissemination, a fact commonly observed in HTLV infection [8,9,52–54]. In Salvador, 32.56% of the family members of the HTLV-1 seropositive women were positive, including children from previous pregnancy [55]. Therefore, the identification of a pregnant woman may be considered just a tip of the iceberg and reinforce the importance of strategies of active surveillance once the infection is diagnosed in an individual.

In Japan, a country with a high HTLV prevalence, a nationwide MTCT prevention program was implemented in 2011. To prevent the vertical transmission, the HTLV-1 seropositive pregnant women are recommended to use three feeding methods: formula feeding, short term breast-feeding (up to 3 months), and feeding with thawed frozen milk. It is noteworthy that infection may occur in the mother after the antenatal screening therefore, the period in which the screening is made may be relevant and it perhaps it should be performed later into the pregnancy. However, the cost of this would be much higher than adding HTLV-1 to the existing screening process. Incident maternal infection during the breast-feeding period has not be considered in this analysis.

Antenatal screening implementation is an important tool to prevent HTLV dissemination. In 1987, the Nagasaki Prefecture in Japan, implemented the ATLL Prevention Program Nagasaki, and the avoidance of breastfeeding by HTLV infected mother resulted in an important reduction of HTLV-1 MTCT from 20.3% to 2.5%, confirming the importance of antenatal screening in disease control [10,56]. Similar results were observed in other Japanese regions [12,57].

It is important to highlight that avoidance of breastfeeding may be harmful, mainly in low-income regions, as early weaning could result in an increased infant mortality and in the number of diarrheal diseases events [58]. Seropositive mothers should have multidisciplinary follow-up to ensure that they receive adequate and necessary information and support.

Although antenatal HTLV-1 screening is performed in some Brazilian’s cities/states, such as Salvador, the city with the highest reported HTLV-1 prevalence, it is not included among the tests that are currently offered for pregnant woman by the Brazilian health system.

Finally, this study showed that the non-implementation of the test in the antenatal routine performed by SUS cannot be justified on the grounds of scarcity of infected individuals, since the national neonatal screening program screens for diseases such as, cystic fibrosis and phenylketonuria that affect approximately 1,250 and 1,225 inhabitants in Brazil respectively [59,60].

Therefore, we conclude that HTLV-1/2 antenatal screening should be implemented in the routine evaluation of pregnant women from Brazil that is offered by the national health system in order to ensure that which is established in the Brazilian Constitution: health as a universal right.
